# Are there differences between officers and ratings on merchant vessels concerning effort–reward imbalance: a cross-sectional maritime field study

**DOI:** 10.1007/s00420-021-01779-8

**Published:** 2021-10-29

**Authors:** Marcus Oldenburg, Hans-Joachim Jensen

**Affiliations:** 1 Institute for Occupational and Maritime Medicine Hamburg (ZfAM), University Medical Center Hamburg-Eppendorf (UKE), Seewartenstrasse 10, 20459 Hamburg, Germany; 2 Flensburg University of Applied Sciences, Flensburg, Germany

**Keywords:** Maritime, Vessels, Seafarer, Overcommitment, Effort–reward imbalance

## Abstract

**Purpose:**

Today, measures to economise in the operation of ships can cause either an effort–reward imbalance or health impairments. The goal of this study was to assess the risk of effort–reward imbalance including overcommitment among officers and ratings on merchant vessels during their assignments and to evaluate lifestyle factors of seafarers as well as the health-promoting conditions on board.

**Methods:**

A study sample of 308 male seafarers was examined during a total of 20 sea voyages on German container ships (participation rate 91.9%).

**Results:**

Only 11 seafarers were identified as having an increased health risk of an effort–reward imbalance (ER ratio > 1). Officers tended to have a higher risk of an elevated ratio than ratings (4.4% vs. 3.1%) and also showed a significantly higher risk of an ER ratio above the median (58.8% vs. 41.8%; *p* = 0.022). Compared to land-based populations, the average overcommitment score of seafarers was high (17.9)—particularly among officers (20.3 vs. 16.5; *p* = 0.031). This corresponded to an elevated risk of overcommitment among officers compared to ratings (OR 2.14; 95% CI 1.78–2.37). This elevated risk remained significant after adjustment for age (OR 2.11; 95% CI 1.76–2.35) and job-related stressors.

**Conclusion:**

Although an elevated risk of effort–reward imbalance was only observed in few seafarers, this study revealed a high prevalence of overcommitment particularly among officers. In the course of time, overcommitment can lead to mental exhaustion. Therefore, shipboard health-promoting conditions need to be optimised.

## Introduction

It is well-known that maritime occupations include a high level of psychophysical strain (Carotenuto et al. 2012; Jespen et al. 2015). This can lead to psychosomatic diseases including burnout syndrome or fatigue (Oldenburg et al. 2013a; Sargent et al. 2017). It cannot be ruled out that work-related stress increases the risk of coronary diseases amongst seafarers. The higher than average age of seafarers, smoking and nutrition habits, their lack of exercise and a high stress burden on board lead to a wide variety of cardiovascular risk factors (Oldenburg et al. 2013b; Baygi et al. 2017). Some of these factors are a result of the combination of working and living on board (Hansen et al. 2005; Apostolatos et al. 2017); for example, the quite often unbalanced and high-fat diet provided, together with a reduction of leisure opportunities and thus too little exercise (Oldenburg et al. 2009; Zyriax et al. 2018). Taking this into account, specificities of food catering on board, leisure opportunities and health education seem to be of great relevance for occupational maritime medicine (Westenhoefer et al. 2018).

A “healthy worker effect” can be postulated for seafarers as they have to undergo medical fitness tests for nautical service every 2 years. Despite this effect, a study by Oldenburg et al. (2008) revealed signs of an increased cardiovascular risk amongst seafarers in comparison to a reference population ashore. Cardiovascular diseases were also identified as one of the most frequent causes of death among seafarers (Jaremin et al. 2003; Ehara et al. 2006).

Today, shipping crews are multinational and heterogeneous (Hansen et al. 2008). They experienced different socialisation patterns in their home countries and the occupational groups vary widely due to their diverse psychophysical challenges. Thus, it is likely that the health status and also the cardiovascular risks differ in respect of the shipboard occupational groups. Furthermore, current seafaring is characterised by increasing economisation in the operation of ships that lead to a growing workload for the crew and can cause either an effort reward imbalance or health impairment. In this context, the question arises about lifestyle factors of the crew (especially concerning sport and nutrition) and the health management on board.

Over the past 10 years, the pooling of multiple data sets into ‘mega-studies’ has accelerated progress in research on stress as a risk and prognostic factor for cardiovascular disease (Kivimäki and Steptoe 2018). In respect of the elevated cardiovascular disease (CVD) risk of seafarers, the Effort–Reward Imbalance (ERI) model was chosen for the present study as its explanatory power concerning work stress parameters, particularly in the case of CVD, has repeatedly been proven (Dragano et al. 2017; Gilbert-Ouimet et al. 2014). The reason for selecting the ERI model without incorporating complementary models (e.g., demand-control, organizational injustice) is that this model is the only one that is not restricted to exclusively extrinsic factors, but also includes an intrinsic component of individual coping (overcommitment). This seems to be particularly relevant in the context of this study. Furthermore, studies published so far focusing on psychological health issues of seafarers on board have never taken the crews’ effort–reward imbalance into account. Besides that, it is common practice to conduct studies dealing with health strains among seafarers ashore (Sargent et al. 2017; Hjarnoe and Leppin 2014). It must be assumed that this setting is not a suitable one to reflect the psychophysical challenges on board a ship in a realistic manner. Therefore, it is desirable to analyse seafarers’ psychophysical stress and health risks during their assignments on board, which generally last for many months.

The goal of this study was to assess the risk of effort–reward imbalance including overcommitment of the officers and ratings in their workplace setting. In addition, lifestyle factors of the seafarers as well as health-promoting conditions on board were evaluated to estimate the need for improved health management on board.

## Methods

### Study sample

**Fig. 1:**
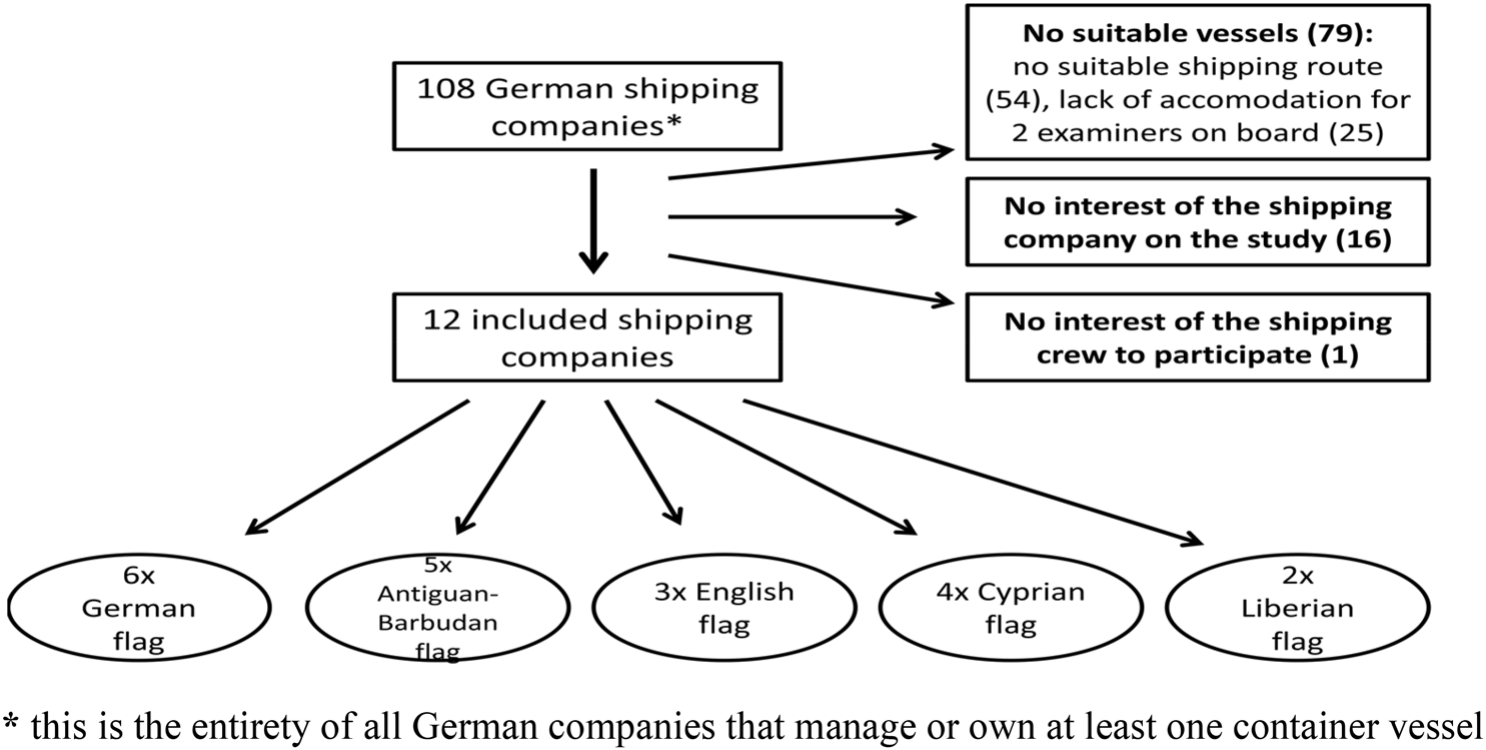
Flow chart of the excluded and included German companies (status 2015). *This is the entirety of all German companies that manage or own at least one container vessel

All 108 German shipping companies that manage or own at least one container vessel were contacted and asked to participate in this study. It is known that the workload aboard depends on the vessel’s shipping route (Baumler et al. 2020; Lochner and Duenser 2018). Particularly container ships with a shipping route in the North or Baltic Sea are subject to high proportions of port handling and only short sea passages, which the seafarers can often use to relax and recover from port-related stress. Thus, only those container vessels with the mentioned shipping routes were included in this study. Due to time restrictions or missing availability of suitable accommodation for the examiners on board, only 12 shipping companies (11.1%) were included in this study (six with at least a second ship) (Fig. 1). Out of these shipping companies, the crews of 20 different container vessels were examined. All shipboard crew members were included in this study, regardless of their occupational group or their origin. The composition of the crews is prescribed by law (Schiffsbesetzungsverordnung (SchBesV) 2013, Federal Ministry of Transport, Building and Urban Development) and thus did not differ significantly between the various ships. Regarding socio-demographic parameters, all officers have a 3-year education at a maritime university and are responsible for management on board—in contrast to the ratings who attend basic courses at maritime schools over a period of 3 years to become able bodied seamen or ship’s mechanics.

In respect of their current flag (6 times German, 3 times English, 5 times Antiguan-Barbudan, 4 times Cyprian and twice Liberian) (Fig. 1), the average construction years of the ships (2006), the predominance of a 3-watch system and the average number of crew members on board (16 persons; range from 11 to 24 persons), there were no differences to the characteristics of the German fleet.

A medically trained scientist accompanied the sea voyages on these 20 container ships and interviewed the whole crew on board. A total of 308 of 335 (participation rate 91.9%) exclusively male seafarers took part in this investigation. The study sample was divided into 194 ratings (63.0%) and 114 officers (37.0%). 79.4% of the ratings and only 7.4% of the officers originated from South Asia, whereas 92.6% of the latter rank came from Europe. An additional differentiation by age resulted in a median of 37 years (range from 18 to 71 years).

Participation in the study was voluntary and the data collection was pseudonymised. All participants gave their written informed consent before taking part in this study. The study was approved by the Ethics Committee of the Hamburg Medical Association (no PV4395).

### Scope of investigation

Objective values of work-related health information were: measurements of the seafarers’ blood pressure and heart rate—as important heart parameters in field studies—carried out before and after two randomly picked shifts on board. A medical doctor measured the blood pressure on both upper arms of the seafarers participating using a manual, single-hand blood pressure monitor with a stable precision manometer (Boso Clinicus II, Jungingen, Germany). During the measurement, the seafarers were in a sitting position and had had a resting phase of at least 10 min before measuring. In cases of significantly differing blood pressure values between the left and the right upper arm or in the pre- and post-shift examination, the measurement was repeated at another time and finally the median calculated. According to the WHO definition, blood pressure is considered to be raised when systolic blood pressure is equal to or above 140 mmHg and/or a diastolic blood pressure equal to or above 90 mmHg (WHO 2020).

Furthermore, the interviewer with seafaring experience conducted a standardised interview on board during the crews’ leisure time at high seas. Due to nowadays commonly multinational shipboard crews, the board language is in general English. Thus, the interview was also performed in English. To avoid possible comprehension problems, the psychologically trained interviewer on board provided direct support on the seafarers' questions. In addition, the interview took place in a confidential setting in the examiner’s single cabin. As a quality criterion, questioning by an interviewer generally enables an immediate completeness check and the elimination of ambiguities (Edwards and Holland 2013; Jamshed 2014). The interview consisted of 2 parts (one standardised questionnaire and questions about health-promoting conditions on board).

#### ***Effort***–***reward imbalance questionnaire (ERI)***

To understand the health risks associated with psychomental and socioemotional work strain, an “Effort–Reward Imbalance” model was developed by Siegrist et al. (1997). This risk score correlates with the cardiovascular risk. According to this model, effort–reward imbalance results from an imbalance between professional effort (exhaustion) and reward received within the work setting (gratification). A ratio > 1 of professional effort divided by reward received indicates a long-lasting stress experience. It should be mentioned that the cut-point 1.0 of the ER ratio has been criticised for methodological reasons, and a standard procedure is also to analyse either continuous data or to define the median or the upper tertile of the distribution as cut-point.

In the present manuscript, the long version of the ERI questionnaire (ERI-L 16 Items. Version 22.11.2012) with a four point Likert answer format is used: (1) strongly disagree, (2) disagree, (3) agree, and (4) strongly agree (Siegrist et al. 2004, 2008). Effort was measured by six items so that the total score measuring extrinsic effort varies between 6 and 24. In analogy to the effort scale, reward was determined by ten four point Likert scaled items resulting in a sum score between 10 and 40. Concerning the seafarers’ effort, additional analyses were performed using the scores of the three sub-scales (esteem, promotion, and security) that provide further meaningful information in theoretical and practical terms (Siegrist et al. 2018; De Jonge and Schaufeli 2002).

In addition, characteristics such as an excessive tendency to push their limits on the job (“overcommitment”) were included in the gratification model. The degree of agreement with the 6 items of the overcommitment scale is determined on the above-mentioned four-point Likert scale. A total sum score is formed from the answers, ranging from 6 to 24 points (Siegrist 2002). A recent study about occupational drivers revealed that high overcommitment scores (cutoff level of 15) were associated with an elevated risk for cardio-vascular diseases (Wei-Te et al. 2019). According to Lehr et al. (2010), a total value of > 16 was regarded as a critical score. Thus, in this study the cutoff level was set at a score value of 16.

The authors of the ERI have repeatedly evaluated the psychometric properties of the Effort–Reward Imbalance questionnaire. The data published document satisfactory internal consistency in terms of Cronbach’s α (usually > 0.70) of the three scales of effort, reward and overcommitment (Siegrist and Montano 2014). In addition, a recent study by Junior et al. (2017) examined the validation of the Effort–Reward Imbalance questionnaire and found a high Cronbach’s alpha for “effort” with 0.78, for “reward” with 0.80 and for “overcommitment” with 0.89. These data were confirmed by other studies (Li et al. 2017). Concerning discriminant validity, significant differences were found in mean scores of effort, reward and overcommitment according to gender, age, socio-economic status, and other sociodemographic characteristics (Wahrendorf et al. 2014).

In respect of the criterion validity, several studies reported convincing sensitivity of the scales to indicate real changes over time (Siegrist et al. 2009). A high score on the ERI scales was associated with elevated risks of poor self-rated health (Siegrist et al. 2009). In total, several studies evaluated the ERI questionnaire as a valid instrument to assess adverse psychosocial work characteristics.

After the effort–reward ratio of the seafarers had been determined, the study population was divided into the groups below and above the median (Siegrist et al. 2003, 2004).

#### Questions about health-promoting conditions on board

After answering the Effort–Reward Imbalance questionnaire the interviewer applied an established maritime ship-specific questionnaire (Oldenburg et al. 2009). In this questionnaire, seafarers were asked about socio-demographic parameters (e.g., age, marital status, existence of children) and job-related aspects (experience at sea (years), average stay on board, average lengths of working time and daily sleep duration). Furthermore, this questionnaire dealt with health-promoting conditions on board—particularly concerning nutrition and exercise during stays on the vessel. As part of this questionnaire, the seafarers had the option to give some information in free text about the established health management on board.

### Statistical analysis

Data analysis was performed with SPSS for Windows (version 20.0, SPSS GmbH Software, Munich, Germany). Continuous variables were presented as mean (± standard deviation (SD)). The Pearson Chi-square test was applied to compare frequencies between groups. After testing for normal distribution, the *t* test was used for the evaluation of differences between groups. The odds ratio (OR) including 95% confidence intervals was calculated with binary logistic regression. The crude OR was first determined and then adjusted for age. Finally, an adjustment of the odds ratio was carried out for the average length of the working day and the daily sleep duration. All indicated *p* values were two-sided, and a *p* value of < 0.05 was deemed statistically significant.

## Results

Regarding socio-demographic parameters, no differences were observed between officers and ratings in age (38.5 vs. 36.6 years; n.s.), in marital status (71% vs. 74%; n.s.) and the existence of children (68% vs. 70%; n.s.). The officers in the present study had longer seafaring experience (15.8 vs. 11.3 years; *p* = 0.002), a longer working day (10.4 vs. 9.3 h; *p* < 0.001) and a shorter daily sleep duration (7.0 vs. 7.6 h; *p* = 0.016). In addition, the average time spent on board varied widely between officers and ratings (4.8 vs. 9.2 months; *p* < 0.001).

### Cardiovascular parameters

While the mean Body Mass Index was 26.4 (± 4.1), slight overweight was observed in the study population (Table 1). No differences in systolic and diastolic blood pressure were measured in the occupational groups on the ship. 18 seafarers (5.8%) showed signs of hypertension (RR ≥ 140/90 mmHg). The resting heart rate did not differ significantly between the two occupational groups. A cross-shift comparison did not reveal any significant changes in the cardiovascular parameters (data not shown).

**Table 1:** Health parameters and smoking habits of the study sample

		Ranks	
		Ratings (194)	Officers (114)
Age, mean (SD)	38.1 (11.1)	37.6 (11.3)	39.5 (10.8)
Body-Mass-Index, mean (SD)	26.4 (4.1)	26.3 (4.2)	25.8 (3.7)
Blood pressure, mean (SD)	123 (10.9)/80 (10.1)	122 (11.4)/79 (10.2)	123 (10.1)/79 (9.2)
Heart rate, *bpm*	81.6 (6.7)	80.8 (6.7)	80.7 (7.2)
Smoking rate, *n* (%)			
Never smokers	158 (51.3%)	102 (52.6%)	56 (49.1%)
Former smokers or current smokers	150 (48.7%)	92 (47.4%)	58 (50.9%)
Pack years, median (min–max)	8.4 (0.1–88)	4.5 (0.1–88)	10.5 (0.2–62)

Even though the occupational groups did not differ in their smoking status (never vs. former/current smoker), the ratings had significantly less pack years (Table 1). There were no differences in alcohol consumption (yes/no) between the professional ranks (*p* = 0.396).

### *Effort*–*reward imbalance questionnaire*

The effort score was distinctly lower than its median within the total study sample. Taking the ERI correction factor into account, the officers displayed a significantly higher effort score than the ratings. The reward score was extremely high in this study, particularly among officers. The latter finding was mainly caused by the officers’ assessment of their job security (*p* = 0.001) and their job promotion. Officers and ratings demonstrated an average effort–reward (ER) ratio of 0.58 and 0.51, respectively. A raised ratio (> 1) corresponding to a higher risk of an effort–reward imbalance was only found in eleven seafarers (4.4% of officers and 3.1% of ratings). The individuals were attributed to below and above the median according to their ER ratio. Officers showed a higher crude risk of an ER ratio above the median than ratings (OR 1.62; 95% CI 1.21–2.78) (Table 2). After adjusting for age, this elevated risk remained significant (OR 1.57; 95% CI 1.18–2.14). Adjustment for the average length of the working day and the daily sleep duration resulted in a decreased but still significant risk of effort–reward imbalance (OR 1.32; 95% CI 1.05–1.89).

**Table 2:** Effort–Reward Imbalance questionnaire in respect of the ranks

	Study sample (308)	Ranks		
		Ratings (194)	Officers (114)	p
Effort–Reward Scores, mean (SD)				
Effort score (range 6–24)	11.3 (2.3)	10.5 (1.1)	12.9 (1.8)	0.015^#$^
Reward score (range 10–40)	35.5 (3.1)	34.5 (2.8)	36.9 (3.2)	n.s.^#^
Job security (range 2–8)	5.2 (1.1)	4.7 (0.7)	6.0 (0.8)	0.001^#^
Esteem (range 4–16)	15.5 (1.3)	15.2 (1.4)	15.7 (1.6)	n.s.^#^
Job promotion (range 4–16)	14.8 (1.1)	14.6 (1.2)	15.2 (1,6)	n.s.^#^
Effort–Reward Imbalance				
Ratio, mean (SD)	0.53 (0.26)	0.51 (0.29)	0.58 (0.18)	0.062^*^
Increased^1^, *n* (%)	11 (3.6%)	6 (3.1%)	5 (4.4%)	n.s.^#^
Median				0.022^#^
< Median	160 (51.9%)	113 (58.2%)	47 (41.2%)	
≥ Median	148 (48.1%)	81 (41.8%)	67 (58.8%)	
Overcommitment				
Score, mean (SD)	17.9 (3.1)	16.5 (2.9)	20.3 (3.4)	0.031^*^
Increased^2^, *n* (%)	181 (58.8%)	107 (55.1%)	74 (64.9%)	0.029^c^

^1^Effort–Reward ratio > 1

^2^Overcommitment score > 16

^*^t test

^#^Chi-square-test

^$^Statistic taking into account the ERI correction factor n.s. = not significant

In respect of the overcommitment scale, the seafarers’ average score was 17.9. The score was higher among officers than ratings (20.3 vs. 16.5; *p* = 0.031). In the comparison of the two occupational groups, officers more frequently had a tendency to push their limits on the job (74 officers (64.9%) and 107 ratings (55.1%); *p* = 0.029). This corresponded to a more than twice as high risk of overcommitment among officers compared to ratings (crude OR 2.14; 95% CI 1.78–2.37). This elevated risk remained significant after adjustment for age (OR 2.11; 95% CI 1.76–2.35). When adjusting for the average length of the working day and the daily sleep duration, a lower risk was observed (OR 1.84; 95% CI 1.74–2.07). Thus, the risk of suffering from both an effort–reward imbalance and overcommitment differed between the two occupational groups on ships (Table 2).

### Health-promoting conditions on board

During the standardised interviews, 74.7% of the seafarer sample stated in free text that health management or tailor-made health education was not established on their ships. 75.8% of the participants would appreciate receiving health information on board; particularly the ratings preferred tailor-made health campaigns implemented by the superiors on board (83.7%).

Furthermore, the crews were asked about nutrition and sport on board:

#### Healthy nutrition

Concerning health-promoting conditions, 115 seafarers (37.3%) were dissatisfied with the nutrition on board, regardless of the rank. Many seafarers complained about too little variety of foods, too much high-fat nutrition and too little fruit, salads and vegetables (Table 3).

**Table 3:** Health-promoting conditions on board

	Study sample (308)	Ranks		Age^1^	
		Ratings (194)	Officers (114)	< 37 years (157)	≥ 37 years (151)
Evaluation of nutrition on board					
Too little variety	90 (29.2%)	51 (26.3%)	39 (34.2%)*	52 (33.1%)	38 (25.2%)
Too much high-fat nutrition	66 (21.4%)	42 (21.6%)	24 (21.1%)	24 (15.3%)	42 (27.8%)**
Too little fruit	62 (20.1%)	46 (23.7%)	16 (14.0%)*	41 (26.1%)	21 (13.9%)*
Too little salads and vegetables	53 (17.2%)	32 (16.5%)	21 (18.4%)	28 (17.8%)	25 (16.6%)
Sport activities^2^ (%)	131 (42.5%)	76 (39.2%)	55 (48.2%)*	72 (45.9%)	59 (39.1%)
Frequency; hours per week (SD)					
Ashore (while on vacation)	5.1 (4.1)	5.1 (4.0)	5.1 (4.3)	5.3 (4.0)	5.1 (4.1)
On board	3.1 (2.9)	2.3 (3.1)	3.7 (2.6)**	3.3 (3.0)	3.0 (2.8)

^1^median of age = 37 years

^2^ “Do you exercise regularly?”

Chi^2^-test: *p < 0.05 and > 0.01; **p < 0.01 and > 0.001

#### Sport activities

A total of 42.5% of the seafarers stated that they did sports regularly. These participants of this study exercised more than 5 h per week ashore and only 3 h on board. The time spent doing sports on board was significantly lower for ratings than for officers (Table 3). 48.1% of the participants indicated a lack of motivation as the main reason to participate less in sport.

## Discussion

According to the seafarers’ statements in the present study, the officers had longer working days (10.4 vs. 9.3 h; *p* < 0.001) and shorter daily sleep durations (7.0 vs. 7.6 h; *p* = 0.016) than ratings, which indicates a higher job-related stress load of this occupational group. In addition, the calculated effort score of the ERI questionnaire was distinctly lower than its median for the total study sample, suggesting that most seafarers regarded their job as not very stressful. The finding that officers had significantly higher effort scores corresponds to their obviously elevated job-related stress load. In this context, it must be considered that officers have a significantly shorter stay on board compared to ratings; this may explain why they also have relatively low effort scores (below the median score).

The reward score was also very high in the total group, so that satisfaction with their work environment can be assumed. Particularly officers seem to be satisfied due to their assessment of their job security (*p* = 0.001) and their job promotion. The significant differences in the job security can be explained by their permanent contracts, while the ratings usually only receive an employment contract for their current ship. Furthermore, compared to ratings, officers have better prospects for job promotion (up to captain or chief engineer of the current vessel).

The mean ER ratio across the study population was 0.53, indicating a low risk of an effort–reward imbalance for seafarers as other studies conducted in land-based settings have shown distinctly higher risk rates. Among health workers the effort‐reward ratio ranges quite widely from 0.47 up to 1.32 and the ER rate from 3.5 to 80.7% (Nguyen et al. 2018). In a study by Wu et al. (2019), 42% of professional drivers were assigned to the higher risk group of an effort–reward imbalance. In the present study, it is possible that the seafarers’ answers about their efforts and demands at work indicate an adaptation to the specific work situation on board or a healthy worker effect. After adjustment for job-related parameters (average length of the working day and daily sleep duration), the risk of an effort–reward imbalance decreased, which is a sign that these job stressors influence this risk. However, the unexpectedly low ER ratio could also be an expression of dissimilation caused by fear of unemployment or social desirability (Eum et al. 2007).

Officers tended to be more often assigned to the group above the median of the ER ratio, which corresponds to the higher job-related stress load among officers mentioned above. Thus, they may have a somewhat elevated risk of an effort–reward imbalance correlating with cardiovascular risk factors. This different risk level between the two occupational groups, however, could be a result of a more pronounced dissimulation among ratings most often originating from South Asia. According to McKay (2007), Filipino seafarers see themselves as the new heroes (“Bagong Bayani”) who take care of their families and have economic significance for their country with their financial transfers. The self-perception as "Bagong Bayani" might have an influence on the dissimulation.

Concerning overcommitment, this study revealed a high average score of 17.9 among seafarers. A study about teachers revealed a critical sum score of 16 (Lehr et al. 2010). Bus drivers—another occupation of the transport industry—showed a median overcommitment value of 13.2 (SD 4.4) (Tse et al. 2007). In total, this study proved the readiness of seafarers to exhaust their personal resources. There are several reasons for this important finding: the crews are involved in the tight shipboard routine for many months without being able to leave the ship to gain inner distance (Jensen et al. 2006). They are mainly focused on the tight working and living situation on board (Haka et al. 2011). Their time at sea is primarily determined by the need to support their family financially (McKay 2007). Furthermore, in the hierarchical structure of ship operation they often experience dominant and less understanding superiors (Sampson and Ellis 2019). In addition, seafarers are aware that safe ship operations require continuous, dedicated and responsible work (Tedesco et al. 2018; Lorenzi et al. 2018). All these circumstances might lead to elevated overcommitment among seafarers.

The risk of overcommitment was more than twice as high for officers as for ratings (OR 2.14; 95% CI 1.78–2.37) regardless of the seafarers’ age, the average length of their working day and their daily sleep duration. The working schedule of the officers demands a high overall work effort due to irregular shifts and especially long working hours (Oldenburg et al. 2009). The willingness to dedicate their time to job-related tasks to an extraordinarily high extent seems to be typical for this working group. The finding of differences regarding the risk of an effort–reward imbalance and overcommitment on board a ship highlights that the different ranks should be taken into account when evaluating workload and strain among the crew (Hansen et al. 2008).

Overcommitment is associated with a higher risk of cardiovascular diseases as a chronic effect. Health limitations, however, can also be caused by potentially harmful individual lifestyle habits (e.g., smoking, high consumption of alcohol, high-fat nutrition, lack of interest in physical activities during leisure time) (Sargent et al. 2017). Despite the physically demanding work on board, the BMI of the study sample averaged 26.4 and indicates slight overweight throughout the whole crew. Therefore, further research is needed focusing on the analysis of specific workload and strain in seafaring.

The high number of smokers in our study sample (48.7%) proves that smoking is still more common for seafarers than for the general population. Tu and Jepsen (2016) already observed a high proportion of smokers/ex-smokers among Danish seafarers (43.1%). Baygi et al. (2017) also described that smoking still counts as one of the most common cardio-metabolic risk factors among seafarers. Although the reason for the high prevalence of smoking among shipboard crews is unclear (e.g., as a consequence of the shipboard stress or due to boredom), there is obviously a need for anti-smoking campaigns. Further studies are required to analyse the benefit of tailor-made health management for the maritime setting. Those studies should motivate the crew to change their lifestyle behaviour, for example through health information campaigns implemented by the superiors on board.

It became obvious during the interview on board that health management or tailor-made health education was not established on three quarters of the ships in our study. It is likely that the crews would perceive these measures as rewards by the shipping company, so that they consequently have a positive effect on the effort–reward balance. According to the present study, effective health promotion measures should encompass the provision of healthy food on board. Furthermore, the crews should be motivated to do more sport (e.g., by organising sport competitions or by providing appealing sport equipment on board). Physical activity is an effective preventive measure for staying in good health and avoiding overweight and musculoskeletal disorders (Baygi et al. 2017); hence it is particularly important for seafarers (Geving et al. 2007; Scovill et al. 2012).

A survey among 570 seafarers employed by a Norwegian shipping company unveiled that 70% did sports ashore at least twice a week, but only 39% did do so on board (Geving et al. 2007). In the present study, the self-reported time of 3 h of physical activity per week (in comparison to 5 h ashore) did not match the examiners’ observations on board. It was obvious that the seafarers generally overestimated their weekly activity doing sport (probably taking all the physically challenging activities into account; regardless of whether they took place during leisure time or working hours). Nevertheless, the reported relation between sports activity ashore and on board can be taken as an indicator that the seafarers’ subjective perception is that of reduced physical activity during their stay on the ships. This observation was especially prominent in ratings.

In total, seafarers are forced to not only spend their working hours but also their leisure time on board. Therefore, health awareness campaigning by superiors on board, a healthy and well-balanced food supply and well-equipped rooms for physical activity appear to be crucial to promoting the psychophysical health of the employees on board (Seppälä et al. 2017).

A strength of the present study is the high participation rate of the nearly 92%. Such high rates are often observed in maritime field studies as most seafarers enjoy the opportunity to distract their thoughts from the often monotonous work and life routine on board. As a limitation of this study, it cannot be excluded that the applied method of face-to-face interviews could have promoted social desirability and that the special working environment of ships’ crews studied may also have contributed to an answering bias. Consequently, the observed low risk of an effort–reward imbalance could in part be the result of seafarers’ worry that disclosure of high work stress might threaten their job security. Furthermore, due to the cross-sectional design, it was not possible to assess long-time effects that may lead to an effort–reward imbalance or to health impairments.

## Conclusions

Although an elevated risk of effort–reward imbalance was only observed among few seafarers, this study revealed a high prevalence of overcommitment particularly among officers. In the course of time, an overcommitment can lead to mental exhaustion. Therefore, the health-promoting conditions on board should be optimised in terms of situation and behaviour prevention (e.g., a balanced, healthy diet and motivation to exercise), which can also have a positive effect on the balance between effort and reward. Furthermore, longitudinal studies on the work-related demands and efforts of shipboard crews are recommended to determine possible chronic health effects.

## Data Availability

The data sets used and/or analysed during the current study are available from the corresponding author on reasonable request.

## Abbreviations

CI: Confidence interval
ER: Effort–reward
ERI: Effort–reward imbalance
OR: Odds ratio
SD: Standard deviation
